# Functional structure of plant communities along salinity gradients in Iranian salt marshes

**DOI:** 10.1002/pei3.10070

**Published:** 2022-03-01

**Authors:** Zeinab Matinzadeh, Jesús López‐Angulo, Adrián Escudero, Sara Palacio, Mehdi Abedi, Hossein Akhani

**Affiliations:** ^1^ Halophytes and C_4_ Plants Research Laboratory, Department of Plant Sciences College of Science School of Biology University of Tehran Tehran Iran; ^2^ Departamento de Biología, Geología, Física y Química inorgánica Universidad Rey Juan Carlos Madrid Spain; ^3^ Department of Environmental Systems Science Swiss Federal Institute of Technology Zurich (ETH) Zürich Switzerland; ^4^ Instituto Pirenaico de Ecología (IPE‐CSIC) Huesca Spain; ^5^ Department of Range Management, Faculty of Natural Resources Tarbiat Modares University Noor Iran

**Keywords:** functional structure, functional trait, Lake Urmia, null model, salt marsh, standardized effect size, trait‐based ecology

## Abstract

Salt marshes are unique habitats between sea or saline lakes and land that need to be conserved from the effects of global change. Understanding the variation in functional structure of plant community along environmental gradients is critical to predict the response of plant communities to ongoing environmental changes. We evaluated the changes in the functional structure of halophytic communities along soil gradients including salinity, in Iranian salt marshes; Lake Urmia, Lake Meyghan, Musa estuary, and Nayband Bay (Iran). We established 48 plots from 16 sites in four salt marshes and sampled 10 leaves per species to measure leaf functional traits. Five soil samples were sampled from each plot and 30 variables were analyzed. We examined the changes in the functional structure of plant communities (i.e., functional diversity [FD] and community weighted mean [CWM]) along local soil gradients using linear mixed effect models. Our results showed that FD and CWM of leaf thickness tended to increase with salinity, while those indices related to leaf shape decreased following soil potassium content. Our results suggest that the variations in functional structure of plant communities along local soil gradients reveal the effect of different ecological processes (e.g., niche differentiation related to the habitat heterogeneity) that drive the assembly of halophytic plant communities in SW Asian salt marshes.

## 1 INTRODUCTION

Salinization is a global degradation process affecting not only soil quality and plant distribution but also the ecosystem services provided by healthy drylands (Flowers et al., 2010; Parida and Das, 2005; Wang et al., 2015). Paradoxically, natural habitats linked to saline soils located between sea or saline lakes and land such as “salt marshes” have been considered of significant importance for nature conservation (Isacch et al., 2006; Milotić et al., 2010; Tabot and Adams, 2013). They are extremely influenced by fluctuations of salinity, which are periodically caused by annual rainfalls, flooding, and inundation (Chapman, 1977; Clarke and Hannon, 1970; Gleason, 1926; Tug et al., 2012). Consequently, salt marshes are fragile and many of them face critical anthropogenic disturbance (Bouchard et al., 2003; Zahran and Willis, 1992). Indeed, direct and indirect anthropogenic factors such as overgrazing, agriculture and intensive irrigation may severely disturb salt marshes and so natural vegetation of these areas (Tug et al., 2012).

Salt marshes are frequently used as study systems to examine plant community structure (Wilson and Whittaker, 1995; Zedler, 1977; Orson and Howes, 1992), as they are colonized by relatively simple plant communities with few dominant species and very low plant diversity (Asri and Ghorbanli, 1997; Cutini et al., 2010; Ghorbanalizadeh et al., 2020; Tug et al., 2012). Generally plant communities in SW and Central Asian salt marshes are dominated by plants specialized to saline soils which their zonation is depending on local topography, existing macroclimate and hydrological conditions. Usually eu‐halophytes such as annual *Salicornia* spp. and C_3_ annual *Suaeda* spp. (*Thero‐Salicornietea* class) occur in highly saline soil near the sea or saline lake, both in inland and littoral marshes. Mangroves (*Avicennia marina* belonging to *Avicennio‐Sonneratietea* class) are restricted to Persian Gulf coasts with tropical macroclimate. Both in temperate and tropical climates, the shrubby and semiwoody stem succulent chenopods (*Halocnemum strobilaceum* and *Halostachys belangeriana*, belonging to *Salicornietea fruticosae* class) dominate the muddy salt flats. The hygro‐halophytic rush and brushwood communities (*Phragmites* spp. and *Tamarix* spp. belonging to *Phragmito‐Magnocaricetea* and *Tamaricetea arceuthoidis* classes) occur in areas where fresh or brackish water inflow from rivers, streams, aquifers, and wetlands. The C_4_ transitional plant formations with species of Chenopodiaceae family are common in moderately saline soils or ruderal salt affected soils as a usually wide zone ending to xerophytic steppes largely dominated by *Artemisia* and *Stipa* species. (Akhani, 2004; Akhani, 2015; Akhani and Mucina, 2015; Djamali et al. 2011; Ghorbanalizadeh et al., 2020).

Several studies on the relationship between vegetation and soil showed that salt concentration in the groundwater, soil salinity, elevation, K, Na, Ca, and Mg content in the soil are strong determinants of soil‐vegetation dynamics in salt marshes (Brewer and Grace, 1990; Cantero et al., 1998; He et al., 2011; Rogel et al., 2000). However, although there is ample information on the differences in plant community composition of salt marshes at the global (Adam, 2002; Simas et al., 2001) and regional scale (Asri, 1998; Ghorbanalizadeh et al., 2020; Niering and Scott Warren, 1980), we have no information on the effect of Na salinity and other soil variables on the functional structure of halophyte communities. Understanding the underlying factors that determine the functional structure of plant communities along local gradients is essential to predict the response of vegetation to different global change drivers and to define conservation programs to protect salt marshes and halophyte communities.

Here, we evaluated the functional structure of plant communities in salt marshes from Iran in relation to salinity and other relevant soil variables. In this regard, we aimed to answer two questions: (i) how does functional plant diversity change with increased soil salinity? (ii) What are the most dominant trait values for certain functional traits in the plant communities occurring along salinity gradients?

## 2 MATERIALS AND METHODS

### 2.1 Study area

**FIGURE 1:**
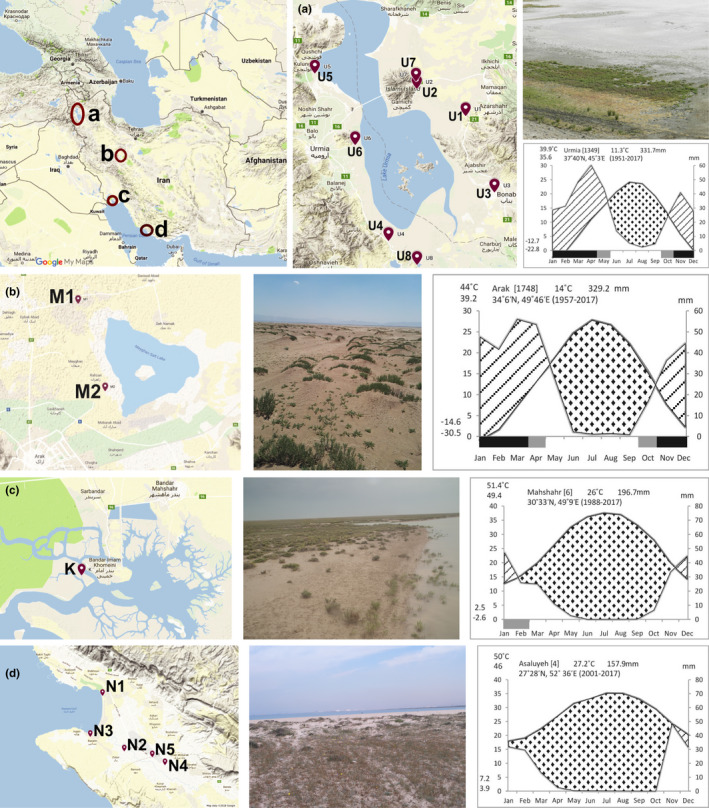
Location of the four studied salt marshes in Iran; Lake Urmia (a), Lake Meyghan (b), Khore Musa (c), and Nayband Bay (d). Inserts show the sites of each sampling area, climatic diagrams (https://www.irimo.ir) for the studied locations, and pictures of the vegetation are also shown

This study was conducted in four salt marshes in Iran: Lake Urmia (NW Iran), Lake Meyghan (Central Iran), Musa estuary and Nayband Bay (South of Iran) (Figure 1; Appendix S1; See Matinzadeh et al. 2019 for further details). All studied salt marshes are dominated by halophytic and salt‐tolerance plants and present local salinity gradients, which lead to a natural zonation of halophytic vegetation. The two first of these salt marshes are inland and both suffer the reduction of water income due to agriculture and unsustainable irrigation management. They are located in a semiarid region with similar bioclimate and so their precipitation is largely similar. The last two salt marshes are coastal with similar bioclimate. Their vegetation is influenced by inundation and tide, which is remarkably different to inland salt marshes (Akhani, 2004; Akhani, 2015; Ghorbanalizadeh et al., 2020).

*Lake Urmia* is known as the largest inland lake in Iran and second largest hypersaline lake in the World (Stone, 2015; ULRP, 2018). Several vegetation zones are identified from the coasts (often a belt of *Salicornia*), muddy high saline plains (*H. strobilaceum*), patches of sedges, C_4_ transitional plant formations, *Tamarix* patches or belts and finally *Artemisia* or ruderal plant communities in undulating hills (Asri, 1998; Asri and Ghorbanli, 1997; Djamali et al., 2008; Ghorbanalizadeh et al., 2020). According to the range of EC (4–182 dS m^−1^) and pH values (7.2–9.4), most soils of this area are saline and alkaline with dominance of sodium and chloride (Asri and Ghorbanli, 1997). Its climate is semiarid continental, which is part of the Irano‐Turanian Xeric Continental (Mxc) bioclimate (Djamali et al., 2011) (Figure 1a).

Lake Meyghan is located in the north of the Arak city, in Markazi Province (Akhani, 2006). The vegetation of this area has an extremely rich halophytic flora consisted of *Halocnemum strobilaceum* in muddy salt flats, *Nitraria schoberi* shrub vegetation (largely cultivated), annual C_4_ dominated chenopods (*Climacoptera* spp., *Bienertia cycloptera*, *Halimocnemis rarifolia*, and *Petrosimonia glauca*), hydrophilous eury‐halophytic rush vegetation (*Phragmites australis*), and *Stipa* steppe (Akhani, 1989). The measured range of EC (0.1–97 dS m^−1^), pH (7.6–9.14) and the formation of saline crusts composed of halite (NaCl), glauberite (Na₂Ca[SO₄]₂), gypsum (CaSO_4_·2H₂O), and calcite (CaCO₃) on the surface of the Meyghan Lake during summer indicate the saline and alkaline soil in this area (Akhani, 1989; Safari‐Sinegani et al., 2017). This area is part of the Irano‐Turanian Xeric Continental bioclimate (Mxc) (Djamali et al., 2011) (Figure 1b).

*Khore Musa (Musa estuary)* is located in the south of Khuzestan Province. Vegetation in this area is dominated by C_3_ communities, consisting of *Salicornia iranica* subsp. *sinus‐persica* and *Suaeda iranshahrii* along the shores followed by C_4_ rich plant zones of *Bienertia sinuspersici*, *Limonium failachicum*, *Suaeda khalijefarsica*, and *S. fruticosa* (Akhani, 2015; Akhani and Deil, 2012; Chatrenoor and Akhani, 2021). Our study site is in the tidal coast of the Musa estuary where the inflow of seawater causes high soil salinity. Sodium and chloride are the main ions in these highly saline soils (Akhani, 2015). This area is part of the tropical desertic (Trd) bioclimate (Djamali et al., 2011) (Figure 1c).

*Nayband Bay* is located near the Asaluyeh industrial zone on the Persian Gulf coasts. Its tidal vegetation includes mostly mangrove forests of *Avicennia marina* followed by *Arthrocaulon macrostachyum* on the high saline shores, *Sporobolus arabicus* on saline sand shores, and end to xerophyte plants on coastal dunes and dry plains (Akhani, 2004). The inflow of seawater in the coastal area and lowlands, the presence of soil layers containing salts and the high transpiration rates caused by strong winds are the main factors causing soil salinity in this area (range of EC = 1.07–10.52 and pH = 8.1–9.8). The presence of sodium and chloride as the main ions along with other ions such as calcium, potassium, magnesium, and sulfate contribute to the high salinity of this area (Akhani, 2015). This area is typical of a tropical xeric (Trx) bioclimate (Djamali et al., 2011) (Figure 1d).

### 2.2 Experimental sampling

We sampled 48 plots from 16 sites (U1‐U8, N1‐N5, K, M1‐2; capital letters indicate the region of origin) in four salt marshes. Sampling sites were selected in the less disturbed areas with almost natural zonation of halophytic vegetation around the lake or sea. Plots were located in areas covered by homogeneous vegetation (avoiding ecotones) with 25 m^2^ surface area in herbaceous vegetation types and 100 m^2^ surface area in shrubby or very open vegetation types in accordance with the most studies with similar vegetation structure (Akhani et al., 2013). The first plots were established on the community observed on the dry lake bed or the sea shore in a given site and continued away from the lake or sea to capture the whole salinity gradient in each region.

Plant cover was visually estimated for each species per plot. Ten mature healthy leaves (or part of photosynthetic shoots of plants with reduced leaves such as *Salicornia*) were randomly sampled from each species in each plot for leaf traits measurements. Five soil samples were collected at ca. 5–15 cm depth in every plot.

Sampling was conducted from early March to October 2015 and 2016, but dates varied among salt marshes depending on plant phenology. Sampling in Lake Urmia was done in spring and summer (May 2015, April 2016 and July 2015), in Khore Musa in autumn (October 2015), in Nayband bay in spring (March 2016) and in Lake Meyghan in summer (June 2016).

### 2.3 Studied species and nomenclature

Studied plants included 188 species collected from 48 plots (Appendix S2). The mean species richness and cover percentage were 10.53 and 8.3, respectively (Appendix S3). Nomenclature is mostly based on Flora Iranica (Rechinger, 1963). Recent generic names were applied for some groups such as Chenopodiaceae (Akhani et al., 2007; Akhani, 2015; Hernández‐Ledesma et al., 2015; Rudov et al., 2020; Chatrenoor and Akhani, 2021).

### 2.4 Functional traits measurement

Six continuous plant traits including plant height (PH), leaf thickness (LT), leaf shape (LS; leaf length (LL)/leaf width (LW)), leaf area (LA), leaf perimeter (LP), specific leaf area (SLA) and leaf dry matter content (LDMC) were measured, obtaining the average of each trait value per species and plot (Table 1). In addition, six categorical traits were included in Appendix S2 to provide more information about study species. These traits, including life history, growth form, and life form were determined according to Pérez‐Harguindeguy et al. (2013), salt‐tolerance category was determined based on Breckle (1990), eco‐morphotypes according to Breckle (1986) and photosynthetic pathway by information available in the literature (Akhani et al., 1997; Akhani and Ziegler, 2002; Osborne et al., 2014; Rudov et al., 2020) (Appendix S2).

**TABLE 1:** Functional traits measured for each species. Abbreviation (Abbr.) and description of their functional relevance, type of variable, units of measurement, and ecological role

Functional trait	Abbr.	Description^a^	Type	Unit	Ecological function^a^
Leaf thickness	LT	Thickness of leaf as an average across the leaf, or at special locations of leaf or tissues	Continuous	mm	Light capture; gas exchange; water retention
Plant Height	PH	The shortest distance between the highest photosynthetic tissue and the ground level	Continuous	mm	Light capture; growth strategy; growth rate; response to climate; competitive ability for light
Leaf Shape	LS	Leaf size; the ratio of Leaf length to leaf width	Continuous	mm	Gas exchange; heat load; response to climate
Leaf Area	LA	One‐sided area of water‐saturated leaf	Continuous	mm^2^	Heat load; water retention; gas exchange; leaf energy and water balance
Specific leaf area	SLA	The ratio of leaf area to leaf dry weight	Continuous	mm^2^ mg^−1^	Photosynthetic capacity; leaf longevity; stress tolerance
Leaf Dry Matter Content	LDMC	The ratio of leaf dry weight to water‐saturated leaf weight	Continuous	mg mg^−1^	Tissue density, physical resistance, stress tolerance, plant nutrient retention and water

^a^ Bernard‐Verdier et al. (2012); Pérez‐Harguindeguy et al. (2013).

The “water saturated‐leaf mass” was measured with a precision balance (Sartorius, TE153S, *d* = 0.001 g) after rehydrating samples for 12 h (and succulent plants for 6 h), and LT was measured by a digital micrometer (Mitutoyo, MDC‐25SB, *d* = 0.001 mm) (Pérez‐Harguindeguy et al., 2013). In detail, we measured thickness of the young succulent stems of stem succulent plants (e.g., *Salicornia*) as LT trait. The scans of water‐saturated leaves were used to calculate LA, LP, LL, and LW using image analysis software ImageJ 1.42q (National Institutes of Health, USA; http://rsb.info.nih.gov/ij). Leaves were subsequently oven dried at 70–75°C for 72 h, and weighed to obtain the “dry leaf mass”. SLA was calculated as the ratio of LA to its dry leaf mass (mm^2^ mg^−1^) (Minden et al., 2012), and LDMC as the dry leaf mass divided by the water‐saturated leaf mass (Vendramini et al., 2002).

### 2.5 Soil chemical analyses

Soil samples were air‐dried, milled in a ball mill (Retsch Mixer MM400) and then dissolved in HCl‐HNO_3_ (9,3) using Microwave Acid Digestion (speedwave MWS‐3^+^, BERGHOF). The filtrated extract solution was used to determine Aluminium (Al), Arsenic (As), Calcium (Ca), Cobalt (Co), Chromium (Cr), Copper (Cu), Iron (Fe), Potassium (K), Lithium (Li), Magnesium (Mg), Manganese (Mn), Molibden (Mo), Sodium (Na), Nickel (Ni), Phosphorus (P), Lead (Pb), Sulfur (S), Silicium (Si), Titanium (Ti), Vanadium (V), and Zinc (Zn) content using inductively coupled plasma‐optical emission spectrometry (ICP‐OES, Varian ICP 720‐ES, analytical services of the Estación Experimental del Zaidín, CSIC, Spain). Total nitrogen and carbon (N and C total) concentrations were measured by an elemental analyzer (Elementar N/CN; VarioMax). Soil pH and electrical conductivity (EC) were measured with a pH/conductivity meter (ORION STAR A215) after diluting with distilled water to 1:2.5 and 1:5 (g: ml), respectively. Soil EC was used to measure and as a surrogate for soil salinity. Also, high Na was used as indicative of high salinity in soil, since Na is a main cation in the studied sites (Akhani, 1989, 2015; Asri and Ghorbanli, 1997). The percentage of gypsum was determined gravimetrically comparing the weight of samples dried at 50 and 105 °C (Porta et al., 1986). Soil carbonate was estimated with a Bernard calcimeter (Bolukbasi et al., 2016) and organic matter was measured through the wet oxidation method (Heanes, 1984). Soil texture was determined with a particle analyzer (Mastersizer 2000, Malvern) (Sochan et al., 2012) (Appendix S4).

### 2.6 Functional structure of plant community

Two complementary indices, community weighted mean (CWM) and functional diversity (FD), were used to evaluate functional structure of plant community (Adler et al., 2014; Batriu et al., 2015; Bernard‐Verdier et al., 2012; de Arruda Almeida et al., 2018). CWM reflects changes in mean functional trait values of the plant communities along salinity gradients (Equation 1) (Garnier et al., 2004; Valencia et al., 2015):

(1) $$\mathrm{CWM}=\sum_{i=1}^{s}a_{\mathrm{ij}}t_{\mathrm{ij}},$$

where *a*_*ij*_ is abundance of the species *i* in the plot *j*, and *t*_*ij*_ is trait value of the species *i* in the plot *j*. FD illustrates the diversity of functional traits in a given community (Equation 2) (de Arruda Almeida et al., 2018; Song et al., 2014; Valencia et al., 2015). For that, we used the mean pairwise distance (MPD):

(2) $$\mathrm{MPD}=\frac{\sum_{i}^{n}\sum_{j}^{n}}{n},\text{where}\;i\neq j,$$

where *n* is the species number in the plots, *δ* the trait distance matrix, and *δ*_*i,j*_ the Gower distance (Gower, 1971; Podani, 1999) of any given single trait between species *i* and *j*.

The correlation of the traits was analyzed by *cor* function in R package “stats” (R Core Team, 2018). LP was excluded before analysis to reduce correlation (>70%). After that, trait values were centered and scaled (Muscarella and Uriarte, 2016) using the *scale* function in Base R (R Core Team, 2018). To compute MPD and CWM independent to local species richness, the functional structure of 34 observed plots (only plots with more than two species were included in the analyses) were compared to 999 random communities derived from null models using an “independent swap” algorithm. We previously tested reshuffling effects on our results, through running three different algorithms of null models: (a) *frequency*, (b) *richness* and (c) *independent swap* algorithms. Because the results of these three null models were similar (Appendix S5), only the results of the ‘independent swap’ algorithm are presented here. Finally, MPD and CWM values of observed and null communities were used to calculate a standardized effect size (SES) by the following equation:

(3) $$\mathrm{SES}=\left(\mathrm{Obs}-{\text{Mean}}_{\text{null}}\right)/{\mathrm{SD}}_{\text{null}},$$

where Obs is the observed value of MPD or CWM in the communities, and Mean_null_ and SD_null_ are the mean and standard deviation of MPD or CWM in the null communities, respectively.

SES of MPD (SES‐MPD) was calculated for six continuous traits in each plot, using the function *ses.mpd* in the R package “picante” (Kembel et al., 2010). SES of CWM (SES‐CWM) was calculated within each community for each six continuous traits separately by the Equation 3.

### 2.7 Statistical analyses

We examined the variation of plant community functional structure along soil gradients using linear mixed effect models (LMMs), such that soil elements were considered as predictors or fixed effects and sites as random effects. By this model, we analyzed the effect of soil variables on trait distribution patterns (SES‐MPD or SES‐CWM; as response factor). To reduce correlation among variables, we checked the correlation of soil variables using *cor* function and selected pH, OM, As, K, Mg, Na, P, Si, and N. After that, we performed a principal components analysis (PCA) using *prcomp* function in R package “stats” selecting the less orthogonal variables with high loading in the first two PCA axes (Appendix S6). As a result, the soil variables selected were K, Mg, Na, and N. Then, VIF scores were used to check for multicollinearity using the *vif* function in the package “car”. Before that, soil parameters were log‐transformed by *log* function in Base R (R Core Team, 2018). The statistical significance of each predictor in the model was determined using likelihood ratio tests (Winter, 2015). Finally, the best model was selected with Na, K, Mg, and N as factors showing the highest contribution to the statistical significance of the model. LMMs were performed using *lmer* function in “lme4” package (Bates et al., 2014) in R i386 3.5.1 (R Core Team, 2018).

## 3 RESULTS

**FIGURE 2:**
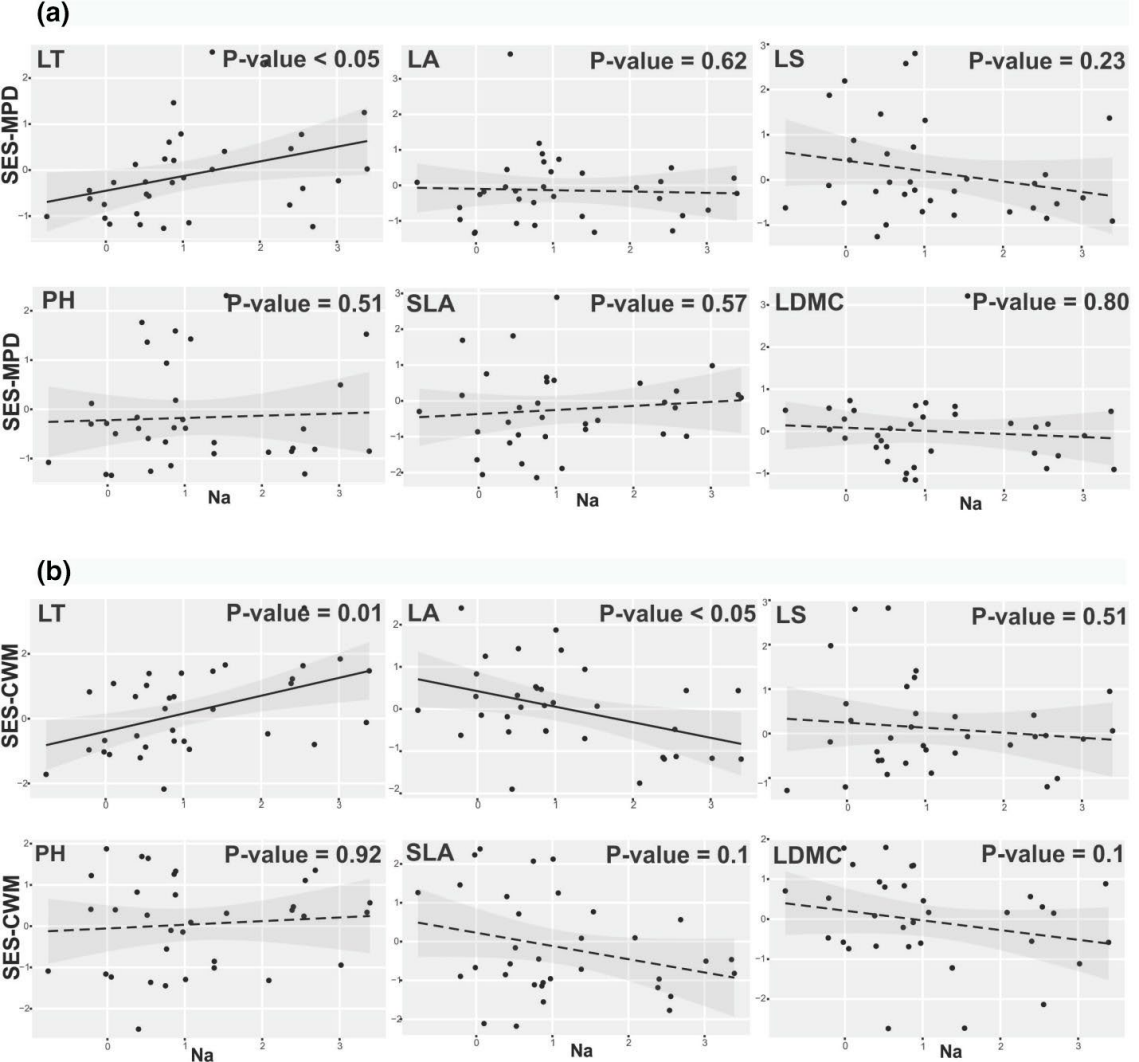
SES‐MPD (a) and ‐CWM (b) of traits along sodium (Na) gradients. Log‐transformed values of soil Na are shown. *P*‐values for every plots show the significance of this relation based on likelihood ratio test. Solid lines represent significant relation while the dashed lines represent non‐significant correlation in the regressions. The 95% confidence intervals for the regressions are shown. LT, leaf thickness; PH, plant height; LS, leaf shape; LA, leaf area; SLA, specific leaf area; LDMC, leaf dry matter content

The SES‐MPD and SES‐CWM of each functional trait showed effective shifts along the soil salinity gradients. LT and LA varied significantly with increasing Na content in the soil, whereas the other four studied functional traits (LS, PH, SLA and LDMC) were independent of soil Na content (Figure 2). SES‐MPD (Figure 2a) and SES‐CWM (Figure 2b) significantly increased with increasing Na content in the soil for LT. In contrast, SES‐CWM of LA significantly decreased along the sodium gradients (Figure 2b).

**FIGURE 3:**
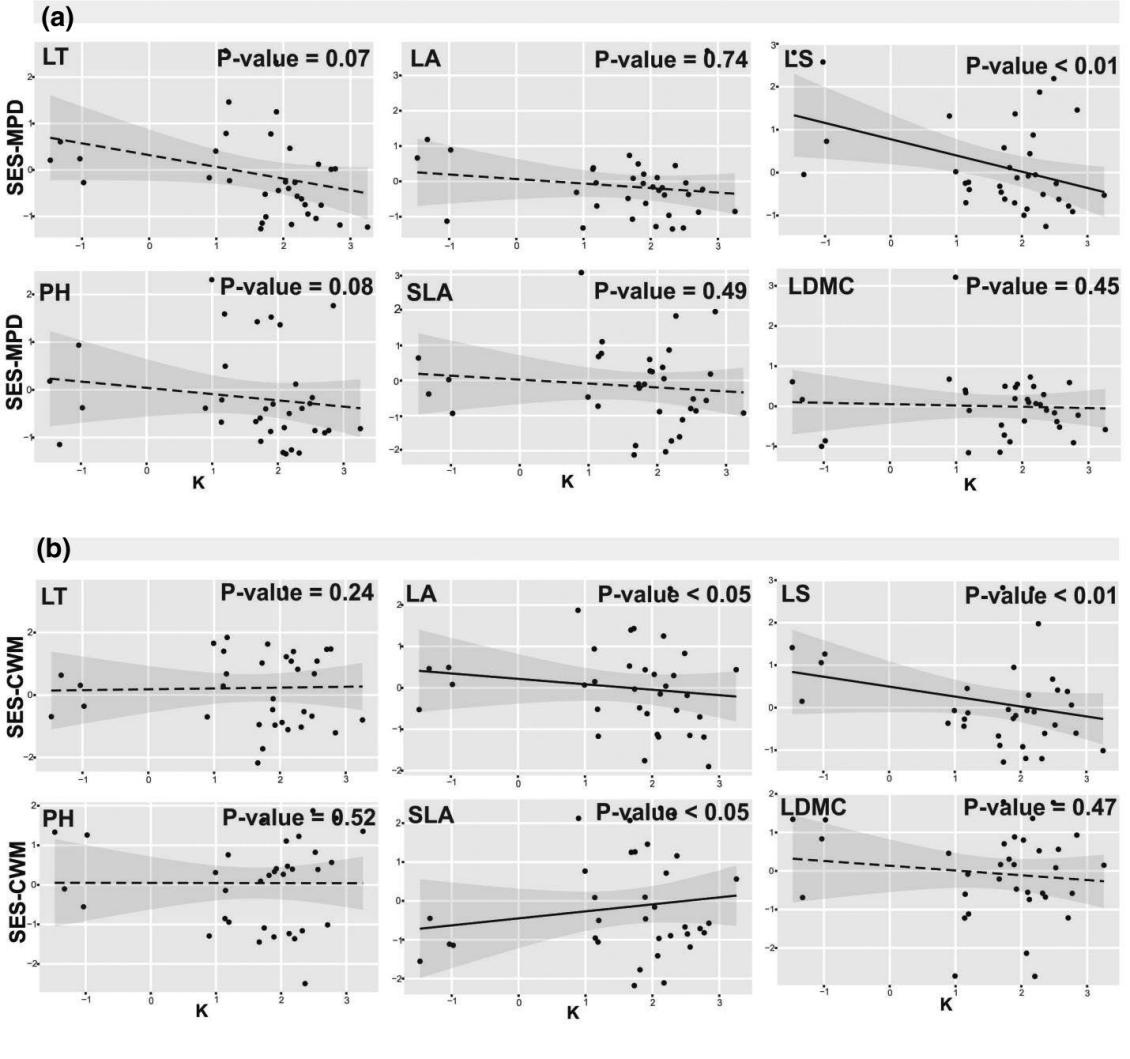
SES‐MPD (a) and ‐CWM (b) of traits along potassium (K) gradients. Log‐transformed values of soil K are shown. *P*‐values for every plots show the significance of this relation based on likelihood ratio test. Solid lines represent significant relation, while the dashed lines represent non‐significant correlation in the regressions. The 95% confidence intervals for the regressions are shown. LT, leaf thickness; PH, plant height; LS, leaf shape; LA, leaf area; SLA, specific leaf area; LDMC, leaf dry matter content

SES‐MPD significantly decreased with increasing soil K for LS (Figure 3a). This trend indicates that LS became less variable in soils with high K concentrations. Moreover, SES‐CWM for LS and LA significantly decreased with increasing soil K (Figure 3b). Conversely, SES‐CWM for SLA significantly increased with increasing K content in the soil (Figure 3b).

**FIGURE 4:**
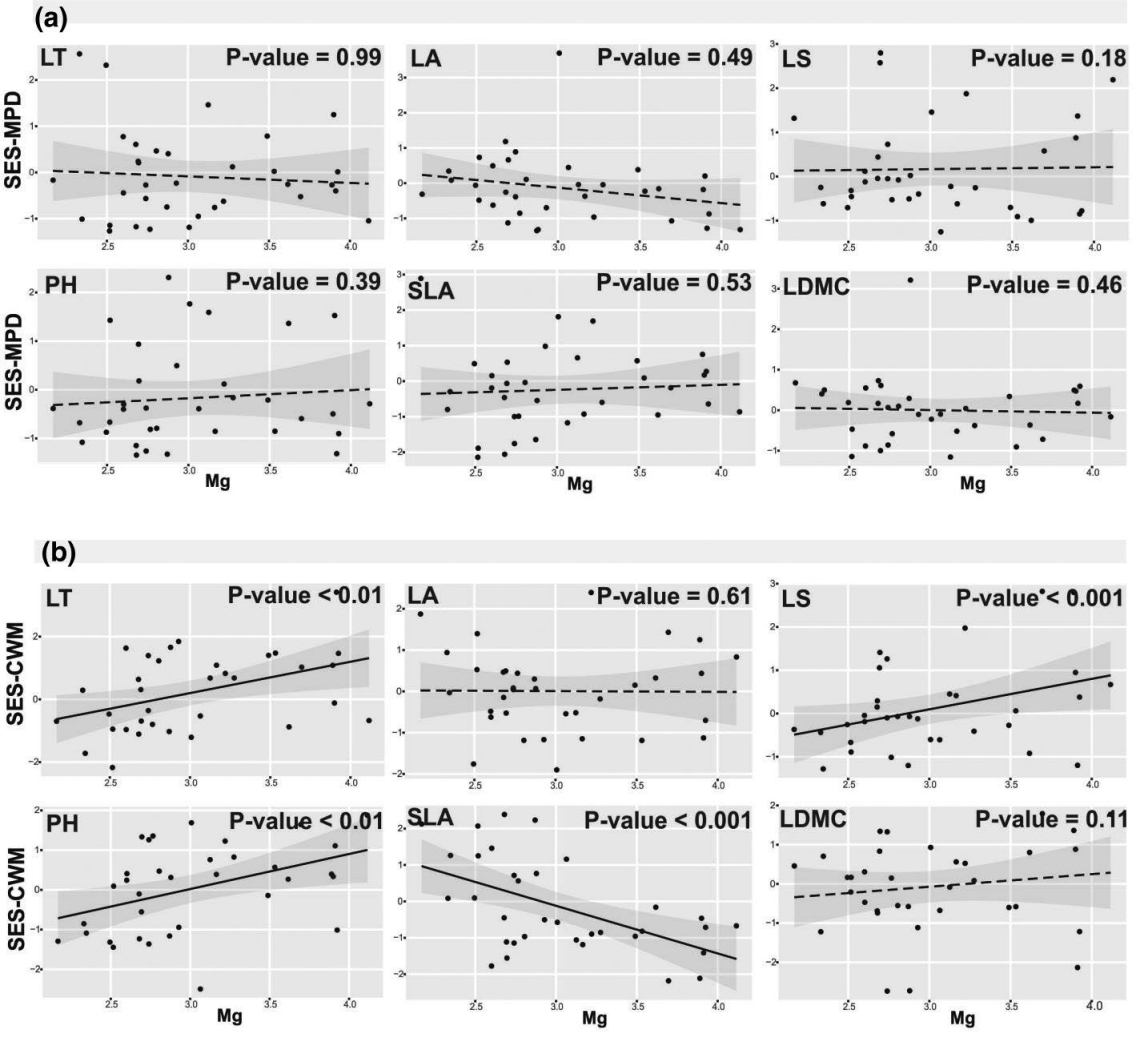
SES‐MPD (a) and ‐CWM (b) of traits along magnesium (mg) gradients. Log‐transformed values of soil mg are shown. *P*‐values for every plots show the significance of this relation based on likelihood ratio test. Solid lines represent significant relation, while the dashed lines represent non‐significant correlation in the regressions. The 95% confidence intervals for the regressions are shown. LT, leaf thickness; PH, plant height; LS, leaf shape; LA, leaf area; SLA, specific leaf area; LDMC, leaf dry matter content

No clear significant patterns were observed for SES‐MPD along soil Mg gradients (Figure 4a). However, SES‐CWM for four of the six functional traits significantly varied along soil Mg gradients (Figure 4b). SES‐CWM for LT, PH and LS increased with increasing Mg content in the soil (Figure 4b). Furthermore SES‐CWM of SLA decreased with increasing Mg content in the soil (Figure 4b).

**FIGURE 5:**
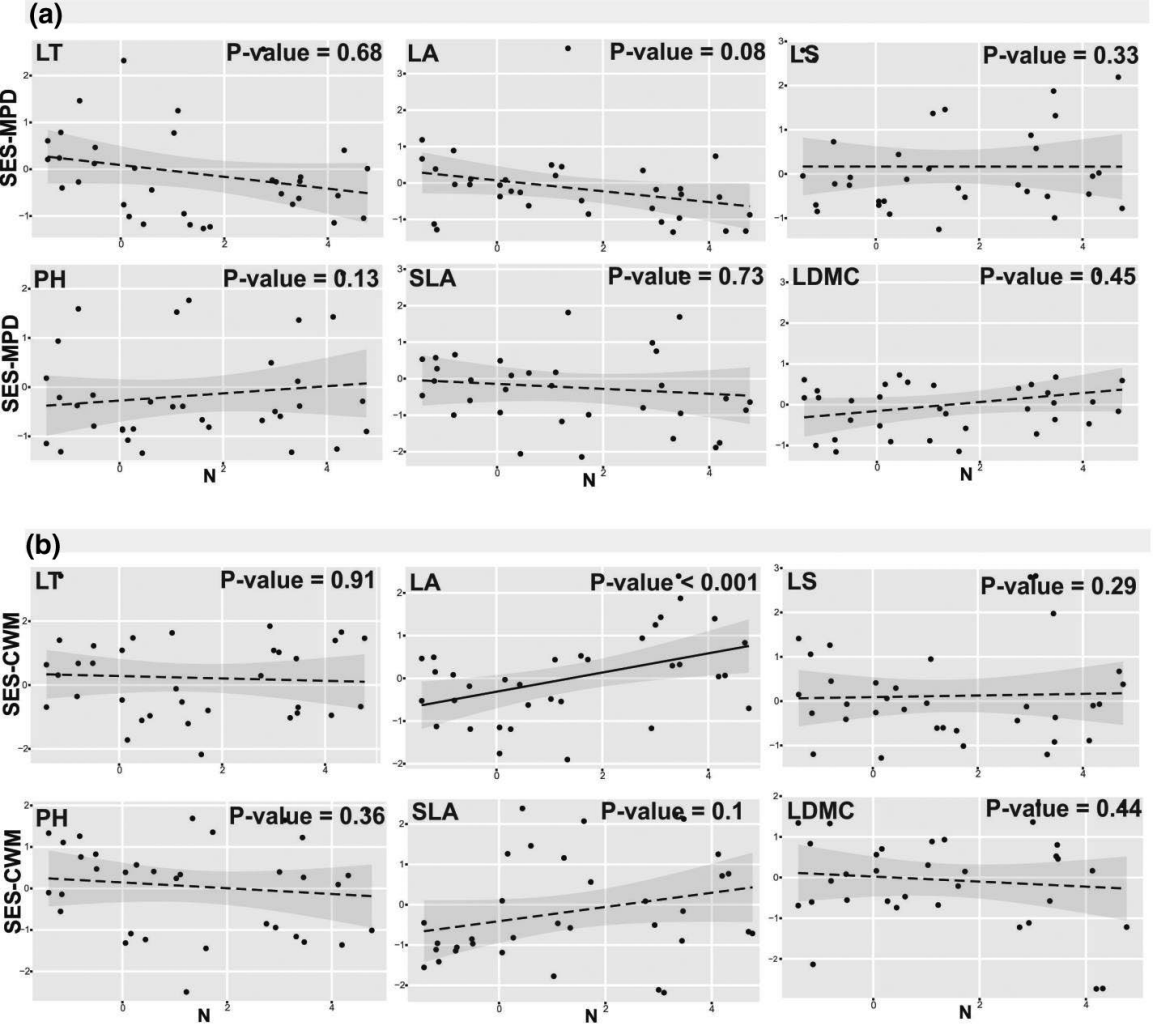
SES‐MPD (a) and ‐CWM (b) of traits along nitrogen (N) gradients. Log‐transformed values of soil N are shown. *P*‐values for every plots show the significance of this relation based on likelihood ratio test. Solid lines represent significant relation, while the dashed lines represent non‐significant correlation in the regressions. The 95% confidence intervals for the regressions are shown. LT, leaf thickness; PH, plant height; LS, leaf shape; LA, leaf area; SLA, specific leaf area; LDMC, leaf dry matter content

There were no significant patterns for SES‐MPD along soil N gradients (Figure 5a). However, we observed an increasing significant trend of SES‐CWM for LA along the soil N gradients (Figure 5b).

## 4 DISCUSSION

MPD and CWM of several functional traits varied along sodium salinity and other soil variables. LT is an indicator of stress tolerance to soil salinity and moisture characterized by leaf succulence (Pérez‐Harguindeguy et al., 2013; Vendramini et al., 2002). The increase of MPD for LT with high Na contents in the soil suggests the coexistence of different functional strategies in high salinity scenarios (i.e., two salt‐tolerance strategies; succulent plants such as *Salicornia iranica* and *Halocnemum strobilaceum* and salt‐recreting plants such as *Atriplex tatarica* and *Aeluropus littoralis*) (Figure 2a; Appendix S3). However, CWM results for LT showed that the most successful strategy in high salinity is that of succulent plants with thick leaves (Figure 2b). So, the increasing pattern of MPD for LT along salinity gradients could be mainly related to habitat heterogeneity within plots, which provides different niches for succulent (e.g., *Salicornia iranica*) and salt‐recreting halophytes (e.g., *Atriplex tatarica*). In detail, succulent C_3_ halophytes of chenopods occur in the wettest and saltiest parts of saline habitats, such as margins of saline rivers, salty lakes, and sea, while salt‐recreting C_4_ halophytes of chenopods mostly occupy the drier parts of saline soil gradient in transition between hygro‐halophytes and xerophytes (Akhani et al., 2003; Frey & Kürschner, 1983). Previous studies have reported a strong heterogeneity in space and time in saline and arid conditions, which can support species with different traits delimiting different niches (de Bello et al., 2006; Ricotta and Moretti, 2011; Scherrer et al., 2018). On the contrary, MPD reduction and low CWM for LT indicated the dominance of thin‐leaved plants (e.g., *Alhagi maurorum*, *Artemisia spicigera*) at low saline condition. Together with thinner leaves, these species may be suited with certain phenological and ecological achievements such as earlier germination, well‐developed root system, vegetative growth, and delayed senescence (i.e., a longer growing season) that enable them to colonize more benign soils with low salinity by avoiding periods of increased salinity (Rozema and Schat, 2013) (Figure 2). In addition, salt‐tolerance plants are the weaker competitor in this condition and so they were excluded. Consequently, our results point at a shift in the dominance strategy along salinity gradients: from two salt‐tolerance strategies including succulent halophytes (e.g., *Suaeda altissima*, *Salicornia iranica*, *Halimocnemis rarifolium*, *Climacoptera crassa*, and *Halocnemum strobilaceum*) and salt‐recreting halophytes (e.g., *Atriplex tatarica*, *Aeluropus littoralis*, and *Frankenia hirsuta*) in the most stressful saline parts of the gradients, to salt‐avoidance strategy characterized by thin leaves and the aforementioned avoidance mechanisms (e.g., *Bromus tectorum*, *Helianthemum salicifolium*, *Sisymbrium septulatum* and *Artemisia spicigera*) in the other edge (Appendix S3) (Brewer and Grace, 1990; Cantero et al., 1998; Ghorbanalizadeh et al., 2020; He et al., 2011; Rogel et al., 2000).

CWM results for SLA in response to shifts in soil K (Figure 3b) indicated a positive relationship between K soil content and high SLA values. SLA reflects plant resource‐use strategy in many environments and relates to plant relative growth rate, photosynthetic efficiency and nutrient conservation strategies (Valencia et al., 2015; Wang et al., 2015; Wilson et al., 1999). Allocation of more resources to photosynthesis and growth is typical in resource‐rich habitats (Thuiller et al., 2010; Wang et al., 2015; Wellstein et al., 2013). Therefore, the dominant plants growing on soils with high K content tend to have high SLA and faster relative growth rates, which could be annual succulent plants. These plants with high SLA can store water in thick tissues of their main photosynthetic organs. Accordingly, our results indicate that these annual succulent plants (e.g., *Suaeda altissima*, *Bienertia cycloptera*, and *Climacoptera lanata*) increased in number of species and abundance in high soil K, and consequently, CWM increased for SLA (Appendix S3).

The presence of succulent plants with high SLA (e.g., *B. cycloptera*, *B. sinuspersici*, *Petrosimonia glauca*, *Suaeda altissima*, *S. khalijefarsica*) along with perennial non‐succulent plants with low SLA (such as *Aeluropus littoralis* and *Frankenia hirsuta*), caused the reduction in the CWM of SLA and the dominance of plants with low SLA in sites with high soil Mg (Figure 4b). The existence of heterogeneous niches in space and time may support the coexistence of these two different strategies (i.e., annual succulent plants and perennial species with low SLA) in soils with high Mg content. The dominance of plants with thin leaves and high SLA (e.g., *Eremopyrum triticeum* and *Hordeum murinum*) in soils with low Mg content (Figure 4b; Appendix S3) could be indicative of a lower investment in storage and defense, a decreased production of fibrous tissue and thinner cell walls and a higher allocation of resources to growth and photosynthesis in these species (Grubb et al., 2015). Furthermore, the opposing pattern of CWM for SLA and LT (Figure 4b) in the soil Mg gradient could be related to more variation and plasticity of SLA than LT (Adler et al., 2014; Gross et al., 2013; Vendramini et al., 2002).

Plant height is an indicator of plant growth form, species position along vertical light gradients, and growth rate (Pérez‐Harguindeguy et al., 2013). A reduction of CWM for plant height or dominance of smaller plants as a direct response to decreasing soil Mg, would be mainly caused for a deficiency of Mg as a soil essential macronutrient (Figure 4b). Conversely, taller plants dominated at the high end of Mg gradient (e.g., *Juncus heldreichianus*, *Stipa hohenackeriana*, *Phragmites australis*; Appendix S3), which is in line with previous findings supporting the view that taller plants might be significantly related to higher availability of some macronutrients such as Mg (Wellstein et al., 2013).

LA and LS can also affect leaf thermal and water conductance since small leaves can help to keep water and leaf temperatures lower in hot and dry conditions (Cornwell and Ackerly, 2009). Low CWM for LA in high soil Na content (Figure 2b) might be due to the adaptation of plants to higher physiological drought which expected under high soil salinity. When saline stress is high, stomata tend to be closed, leading to high leaf temperature and high damage to large leaves (Cornwell and Ackerly, 2009). Our results, similar to previous studies, showed that plants with high LA generally tend to grow in milder saline habitats, because their photosynthetic rates would be high in this condition (Cornwell and Ackerly, 2009; Gross et al., 2013). Low CWM for LA and LS or the dominance of small‐leaved plants in low soil Mg and N, respectively, could be due to macronutrient (i.e., Mg and N) deficiencies (Figures 4b and 5b) (Trubat et al., 2006; Watson, 1947).

High MPD of LS in plots with low soil K may indicate the high niche differentiation in heterogeneous environments between functionally different plants with similar responses to low K and also high Na content in the soil (i.e., large‐elongated non‐succulent leaves, e.g., *Bolboschoenus glaucus* and small‐scaly succulent leaves, e.g., *Caroxylon imbricatum*) (Figure 3a; Appendix S3). Furthermore, the high CWM for LA and LS in low soil K (Figure 3b) indicates the most successful strategy is that of large and long leaved plants such as *Bolboschoenus glaucus*, which are distributed in margins of saline and moderately saline lakes, salty and brackish swamps (Akhani and Ghorbanli, 1993). These results may be explained by the antagonism effect of K and Na in the soil, especially in sodic or saline‐sodic soils, where that high Na content and saline stress is linked to low soil K (Matinzadeh et al., 2013; Wakeel, 2013).

## 5 CONCLUSION

Our results demonstrate that the functional structure of plant communities in Iranian salt marshes may change along sodium and associated nutrient content gradients. We found that the increase of MPD and CWM for LT along soil Na gradients would be related to niche differentiation in heterogeneous environments and dominance of succulent plants in high saline soil. In addition, MPD and CWM of LS decreased along soil K gradients and the plants with small‐elongated leaves are dominant in high K content in the soil. Furthermore, the reduction of CWM for LA along soil Na gradients indicates the plants with small leaves are the most successful plants in high Na content in the soil, while the increase of CWM for LA towards soil N availability would be mainly related to success of plants with large leaves and high photosynthetic rates in high macronutrient availability. We conclude that the variations in functional structure of plant communities along environmental gradients can display some ecological processes such as niche differentiation related to habitat heterogeneity, which can influence the assembly of halophyte communities in Iranian salt marshes.

## CONFLICT OF INTEREST

The authors have no conflict of interests to declare.

## AUTHOR CONTRIBUTIONS

M.A., Z.M., and H.A. conceived of the research idea; Z.M. and H.A. collected data; Z.M. and S.P. performed soil chemical analysis; Z.M., J.L.A., and A.E. performed statistical analysis; Z.M. wrote the paper; all authors discussed the results and commented on the manuscript.

## Supporting information


Appendix S1
Appendix S2
Appendix S3
Appendix S4
Appendix S5
Appendix S6.

## Data Availability

The data that supports the findings of this study are available in the supplementary material of this article.
